# Prevalence and Socio-Professional Determinants of Organic Psychosyndrome among Workers Exposed to Organic Solvents

**DOI:** 10.1192/j.eurpsy.2026.11519

**Published:** 2026-07-31

**Authors:** J. Turki, N. Kotti, F. Dhouib, N. Remadi, M. Hajjaji, K. Jmal Hammami

**Affiliations:** 1 Intercompany Occupational Health Service, Calais, France; 2 Occupational medecine Department, Faculty of medecine of Sfax, Sfax, Tunisia

## Abstract

**Introduction:**

Occupational exposure to organic solvents (OS) has been associated with a range of neuropsychiatric effects, including the development of Organic Psychosyndrome (OPS). OPS is characterized by cognitive, emotional, and behavioral disturbances that may significantly impair work performance and quality of life. Despite its potential impact, the condition often remains underdiagnosed in occupational health settings.

**Objectives:**

This study aimed to determine the prevalence of OPS and to investigate the socio-demographic and occupational factors associated with its occurrence among employees exposed to organic solvents.

**Methods:**

Cross-sectional, descriptive and analytical study including plastics industry workers exposed to SO during the period from June 1 to August 30, 2019. The assessment of OPS was carried out using a questionnaire inspired by the Swedish version of Q16 questionnaire.

**Results:**

During the study period, we interviewed 130 workers with a mean age of 35±8.4 years and a sex ratio of 1.28. A history of respiratory allergies was present in 15.3% of cases. Regarding occupational characteristics, 63% of the employees interviewed had less than 10 years of professional experience. 47.7% of the study population were unskilled workers. The mean occupational exposure to OS was 6±4 years. The highest OS exposure rate was for acetone and styrene (85.2%). Collective prevention was mainly based on natural ventilation (63.1%). Daily wearing of respiratory masks was only present in 22.3% of cases. The prevalence of OPS in our study was 21.5%. In the analytical study, OPS was associated with female gender (p=0.04) and respiratory allergy (p=0.03). Young age (<30 years) appeared to decrease the risk of OPS (p=0.005). The risk of OPS was increased by a duration of exposure to OS longer than 10 years (p=<10-3), daily exposure (p=0.04), and direct exposure to OS (p=0.017). Regarding prevention methods, vacuuming at source (p=0.04) and the daily wearing of cartridge respirators (p=0.03) reduced the risk of OPS.

**Conclusions:**

Exposure to OS is a real risk for exposed employees. This risk is even greater when the frequency of handling and duration of exposure are high. Strengthening technical and medical prevention is the only way to reduce the neurological effects of occupational exposure to OS.

**Disclosure of Interest:**

None Declared

